# An Activity Tracker–Guided Physical Activity Program for Patients Undergoing Radiotherapy: Protocol for a Prospective Phase III Trial (OnkoFit I and II Trials)

**DOI:** 10.2196/28524

**Published:** 2021-09-22

**Authors:** Franziska Hauth, Barbara Gehler, Andreas Michael Nieß, Katharina Fischer, Andreas Toepell, Vanessa Heinrich, Inka Roesel, Andreas Peter, Mirjam Renovanz, Andreas Hartkopf, Andreas Stengel, Daniel Zips, Cihan Gani

**Affiliations:** 1 Department of Radiation Oncology University Hospital Tübingen Tuebingen Germany; 2 Edwin L Steele Laboratories for Tumor Biology, Department of Radiation Oncology Massachusetts General Hospital Harvard Medical School Boston, MA United States; 3 Department of Sports Medicine University Medicine Tübingen Tuebingen Germany; 4 Institute for Clinical Epidemiology and Applied Biostatistics University Hospital of Tübingen Tuebingen Germany; 5 Department of Diagnostic Laboratory Medicine Institute for Clinical Chemistry and Pathobiochemistry University Hospital of Tübingen Tuebingen Germany; 6 Department of Neurology & Interdisciplinary Neuro-Oncology University Hospital Tübingen Hertie Institute for Clinical Brain Research Tuebingen Germany; 7 Department of Obstetrics and Gynecology University Hospital Tübingen Tuebingen Germany; 8 Department of Psychosomatic Medicine and Psychotherapy Medical University Hospital Tübingen Tuebingen Germany; 9 Comprehensive Cancer Center Section Psychooncology University Hospital Tuebingen Tuebingen Germany; 10 Charité Center for Internal Medicine and Dermatology Department of Psychosomatic Medicine Charité-Universitätsmedizin Berlin Berlin Germany; 11 Freie Universität Berlin Humboldt-Universität zu Berlin Berlin Institute of Health Berlin Germany; 12 German Cancer Research Center Heidelberg and German Consortium for Translational Cancer Research Partner Site Tübingen Tuebingen Germany

**Keywords:** cancer, fatigue, physical activity, quality of life, activity tracker, exercise program, radiotherapy, digital health

## Abstract

**Background:**

The positive impact that physical activity has on patients with cancer has been shown in several studies over recent years. However, supervised physical activity programs have several limitations, including costs and availability. Therefore, our study proposes a novel approach for the implementation of a patient-executed, activity tracker–guided exercise program to bridge this gap.

**Objective:**

Our trial aims to investigate the impact that an activity tracker–guided, patient-executed exercise program for patients undergoing radiotherapy has on cancer-related fatigue, health-related quality of life, and preoperative health status.

**Methods:**

Patients receiving postoperative radiotherapy for breast cancer (OnkoFit I trial) or neoadjuvant, definitive, or postoperative treatment for other types of solid tumors (OnkoFit II trial) will be randomized (1:1:1) into 3-arm studies. Target accrual is 201 patients in each trial (50 patients per year). After providing informed consent, patients will be randomized into a standard care arm (arm A) or 1 of 2 interventional arms (arms B and C). Patients in arms B and C will wear an activity tracker and record their daily step count in a diary. Patients in arm C will receive personalized weekly targets for their physical activity. No further instructions will be given to patients in arm B. The target daily step goals for patients in arm C will be adjusted weekly and will be increased by 10% of the average daily step count of the past week until they reach a maximum of 6000 steps per day. Patients in arm A will not be provided with an activity tracker. The primary end point of the OnkoFit I trial is cancer-related fatigue at 3 months after the completion of radiotherapy. This will be measured by the Functional Assessment of Chronic Illness Therapy-Fatigue questionnaire. For the OnkoFit II trial, the primary end point is the overall quality of life, which will be assessed with the Functional Assessment of Cancer Therapy-General sum score at 6 months after treatment to allow for recovery after possible surgery. In parallel, blood samples from before, during, and after treatment will be collected in order to assess inflammatory markers.

**Results:**

Recruitment for both trials started on August 1, 2020, and to date, 49 and 12 patients have been included in the OnkoFit I and OnkoFit II trials, respectively. Both trials were approved by the institutional review board prior to their initiation.

**Conclusions:**

The OnkoFit trials test an innovative, personalized approach for the implementation of an activity tracker–guided training program for patients with cancer during radiotherapy. The program requires only a limited amount of resources.

**Trial Registration:**

ClinicalTrials.gov NCT04506476; https://clinicaltrials.gov/ct2/show/NCT04506476. ClinicalTrials.gov NCT04517019; https://clinicaltrials.gov/ct2/show/NCT04517019.

**International Registered Report Identifier (IRRID):**

DERR1-10.2196/28524

## Introduction

Radiotherapy is a key treatment modality for the curative treatment of various tumor entities. Despite continuous technical improvements in facilitating increasingly precise dose delivery, side effects are inevitable in many cases [1]. Among patients with breast cancer, cancer-related fatigue (CRF) is the most frequent side effect reported during and after postoperative radiotherapy [2]. Among patients with other tumor sites that were treated with preoperative or definitive radiochemotherapy, CRF has also been frequently reported. However, local acute and late side effects of treatment can cause further impairments in health-related quality of life (HRQoL) [1]. In addition, sedentary behavior during preoperative radiotherapy can have a negative impact on the postoperative course of a patient (eg, in terms of recovery and wound complications). The beneficial effect that physical activity has on patients with cancer has been clearly established over recent years and is supported by many clinical studies [3-5]. Moreover, many international organizations have updated their recommendations to include exercise as an important part of cancer therapy (ie, exercise before, during, and after cancer therapy) [6-8]. Guidelines for the inclusion of exercise in oncologic treatments for patients with cancer have been published recently [9]. In this context, physical activity training can be implemented at different time points during treatment. The goal of prerehabilitation is to improve the fitness of patients prior to undergoing a major medical intervention, such as cancer surgery [10]. Faster recovery, fewer wound complications, and improved HRQoL have been exhibited by patients who have participated in a prerehabilitation program [11-13]. In patients with breast cancer, low levels of physical activity are associated with a 22% higher risk of breast cancer mortality [14]. Furthermore, a recent meta-analysis has shown that physical activity during oncological treatments is a highly effective measure for reducing CRF [15]. Additionally, in patients that survive cancer after oncological treatment, a higher level of physical activity is associated with improved HRQoL and, in some studies, even improved cancer-specific survival [14,16]. Although the pathomechanisms leading to CRF remain unclear to date, previous studies have suggested a close relationship between CRF and proinflammatory pathways, including pathways that lead to increases in levels of interleukins [17,18].

Activity trackers, which are also called *wearables*, have been used by a growing population to record physical activity and biodata, such as pulse and sleep patterns. Although fitness wristbands were initially considered purely as lifestyle products, in recent years, the scientific benefits of these products have increasingly come to the fore [17-19]. However, no prospective trials that include activity trackers as part of patients’ radiotherapy have been conducted to assess these trackers’ efficacy in reducing CRF and improving HRQoL.

In the presented OnkoFit studies, we aim to investigate whether an activity tracker–based fitness program can reduce CRF and improve the quality of life and preoperative health status of patients undergoing radiotherapy.

## Methods

### Study Setting and Participants

**Figure 1:**
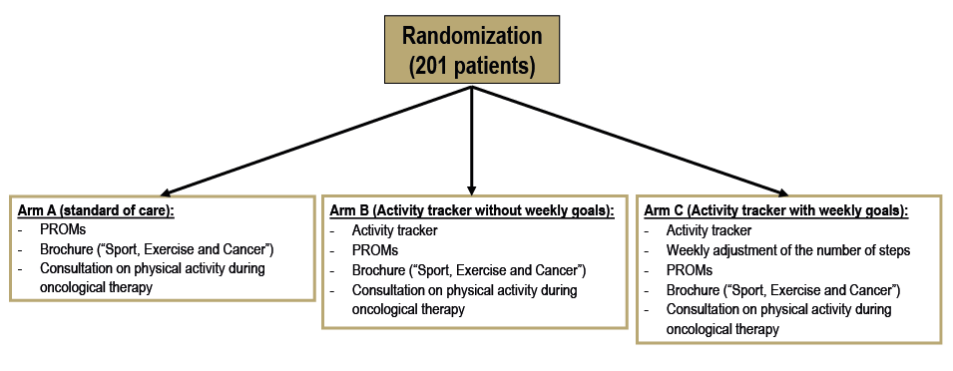
Overview of study arms of the OnkoFit I and OnkoFit II trials. In each study, patients will be randomized (1:1:1; 67 patients per arm) into the 3 arms after providing informed consent. PROM: patient-reported outcome measures.

The OnkoFit trials are two independent, single-center, randomized prospective phase III trials that have been certified by the working group radiological oncology of the German Cancer Society. After providing informed consent, patients will be randomized to a standard arm (arm A) or 1 of 2 interventional arms (arm B and C) in a 1:1:1 ratio (Figure 1). All patients will be recruited by the Department of Radiation Oncology at the University Hospital Tuebingen.

The OnkoFit I trial is exclusively designed for patients with breast cancer, while the OnkoFit II trial is open to patients undergoing preoperative, definitive, or postoperative radiotherapy for various tumor sites. The separation of the tumor entities in two different trials allows for two different end points. Although the end point of the OnkoFit I trial—fatigue at 3 months after radiotherapy—has been evaluated in a pilot trial that was conducted in our department [2], it was determined that for patients undergoing neoadjuvant radiochemotherapy, this end point might be too early, especially for patients who undergo surgery after neoadjuvant radiochemotherapy.

**Figure 2:**
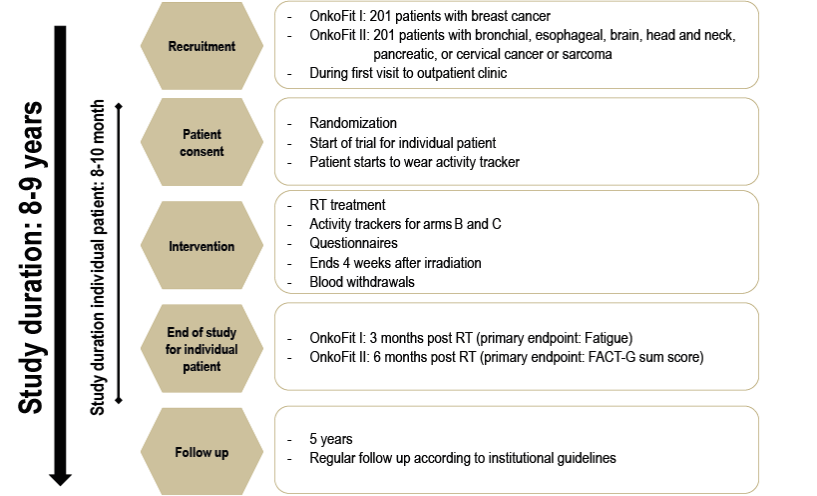
Overview of the study design and expected duration. FACT-G: Functional Assessment of Cancer Therapy-General; RT: radiotherapy.

A summary of key inclusion and exclusion criteria is provided in Textbox 1. We anticipate the recruitment of 50 patients per year in each trial. The course of the study is displayed in Figure 2.

**Inclusion and exclusion criteria of the OnkoFit trials.**

**Inclusion criteria**

- OnkoFit I trial
  Informed consent
  Age>18 years
  Histologically confirmed breast cancer
  Easter Cooperative Oncology Group score of 0-2
  Indication for postoperative radiotherapy after breast-conserving surgery or mastectomy
- OnkoFit II trial
  Informed consent
  Age>18 years
  Diagnosis of lung cancer, esophageal cancer, brain tumors, head and neck tumors, pancreatic cancer, rectal cancer, sarcoma, and uterus cervix cancer
  Easter Cooperative Oncology Group score of 0-2
  Indication for preoperative, definitive, or postoperative radiochemotherapy
  Planned duration of treatment of at least 4 weeks

**Exclusion criteria**

- OnkoFit I trial
  Participation in other interventional trials
  History of using an activity tracker
  Pregnancy
  Recent cardiovascular events (stroke, myocardial infarction within the last 6 months, and a cardiac insufficiency New York Heart Association grade of >I)
  Easter Cooperative Oncology Group score of 3-4
  Comorbidities with impairments of mobility, such as paraplegia
- OnkoFit II trial
  Participation in other interventional trials
  History of using an activity tracker
  Pregnancy
  Recent cardiovascular events (stroke, myocardial infarction within the last 6 months, and a cardiac insufficiency New York Heart Association grade of >I)
  Easter Cooperative Oncology Group score of 3-4
  Comorbidities with impairments of mobility, such as paraplegia

### Treatment Groups and Interventions

After providing informed consent, patients will be included into either the OnkoFit I trial or OnkoFit II trial and randomized into 1 of the 3 arms of the studies (Figure 1). Both studies aim to include 201 subjects each.

#### Arm A: Standard of Care

**Figure 3:**
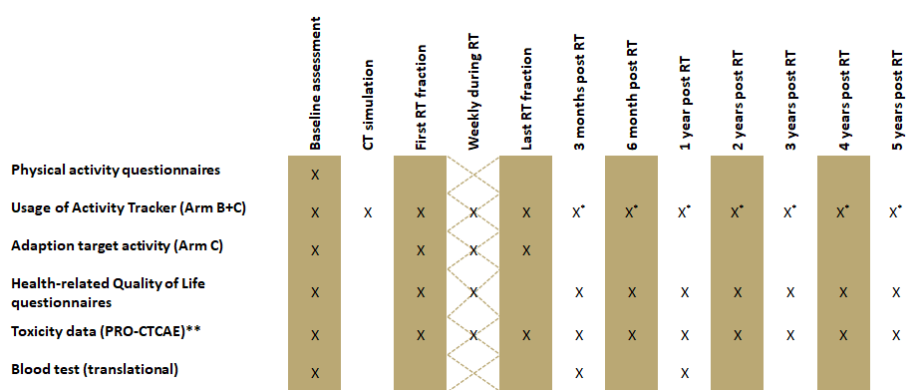
Overview of the procedure plan and follow-ups for after radiotherapy. The quality-of-life questionnaires include the Patient Health Questionnaire-8, Functional Assessment of Cancer Therapy-Breast, Functional Assessment of Chronic Illness Therapy-Fatigue, and European Organisation for Research and Treatment of Cancer Quality of Life-Cancer 30 questionnaire. *The use of the activity tracker after intervention completion is based on patients’ preferences. **PRO-CTCAE items were selected based on the anatomical region treated. PRO-CTCAE: Patient-Reported Outcomes Common Terminology Criteria for Adverse Events; RT: radiation therapy.

In this arm, patients will be advised to conduct at least 1.5 hours of moderate physical activity or 75 minutes of strenuous physical activity per week. Patients will receive an educational patient brochure (written in the German language) about the rational and potential benefits of physical activity during cancer treatment. Activity trackers will not be provided to the patients (Figure 3).

#### Arm B: Activity Tracker Without Weekly Goals

As in arm A, patients in arm B will be advised to conduct 1.5 hours of moderate physical activity or 75 minutes of strenuous physical activity per week and will receive the previously mentioned patient brochure. In addition, patients will be given an activity tracker. They will be instructed to wear it throughout the day and document the daily step count in a patient activity diary, which will be provided to each patient. No instructions will be provided with regard to daily or weekly goals for step counts (Figure 3).

#### Arm C: Activity Tracker With Weekly Goals

As in the other arms, patients will be counseled about conducting 1.5 hours of moderate physical activity or 75 minutes of strenuous exercise and will receive the patient brochure. The same activity tracker as that in arm B will be given to patients. However, in arm C, patients will receive weekly goals for their average step counts during treatment. The baseline step count will be assessed from the time of informed consent provision until the time of computed tomography simulation (computed tomography scans of patients will be taken prior to treatment to allow for radiation treatment planning). This period usually amounts to approximately 7 days. The patients will document daily step counts in a patient activity diary. At the time of computed tomography simulation, the mean daily step count will be calculated; this will constitute the basis for the upcoming week’s goal for step counts. If the average step count is 6000 or higher, the new goal will be to not fall under this value. If the average step count is less than 6000, the new goal will be calculated by increasing the past week’s step count average by 10%. If, for instance, the average step count is 3000 steps, the new target will be 3300 steps. The last change to the target step count will take place during the last week of radiotherapy. The highest possible target step count is 6000. This means that an average of 5800 steps in 1 week will result in a new goal of 6000 steps. We chose this 6000-step threshold based on a literature search we conducted, which suggested that a sedentary lifestyle is defined by threshold [16] (Figure 3).

In all arms, blood samples will be taken at baseline, at the end of radiotherapy, and at 6 months after the end of treatment. The activity trackers that will be used in this trial are commercially available accelerometers.

### Clinical End Points and Instruments Used

#### OnkoFit I Trial

For the OnkoFit I trial, the primary end point will be fatigue, which will be scored according to the Functional Assessment of Chronic Illness Therapy-Fatigue (FACIT-F) subscale. The end point will be assessed 3 months after the completion of radiotherapy. The FACIT-F questionnaire, which consists of 40 questions, was validated to assess fatigue in patients with cancer among our own patient cohort in a pilot project, which was conducted prior to the OnkoFit trials [2], as well as in many other studies [20,21]. The secondary end points will include treatment compliance, patient-reported acute and late toxicity, disease-free survival, and overall survival.

#### OnkoFit II Trial

For the OnkoFit II trial, the primary end point will be the overall HRQoL, which will be assessed by the Functional Assessment of Cancer Therapy-General sum score. This end point will be measured 6 months after the end of treatment in order to account for possible surgical interventions and possible recovery thereafter. In addition to the secondary end points listed for the OnkoFit I trial, we will assess the frequency of unplanned inpatient treatments for patients planned for ambulatory treatments and the frequency of postoperative complications (which will be assessed by using the Clavien-Dindo classification system) [22] in patients receiving preoperative radiotherapy.

Treatment-related toxicities will be scored by using the German translation of the Patient-Reported Outcomes Common Terminology Criteria for Adverse Events (PRO-CTCAE), which were developed by the National Cancer Institute. Briefly, the assessment of the severity and frequency of symptoms will be based on a 5-tier scale that ranges from “none” to “very severe” or from “never” to “almost always.” Depending on the treated site (breast cancer: 8 questions; head and neck: 27 questions; thoracic and abdominal lesions: 21/24 questions; brain cancer: 20 questions; sarcoma: 14 questions), specific sets of questions were created. The patient-reported outcome measurement questionnaires for patients with head and neck cancer as well as patients with thoracic and abdominal tumor lesions have been used in our department [1]. The selection of PRO-CTCAE items was based on our long-term experience with the most frequent side effects that occur during and after radiotherapy for treated regions. Depression will be assessed with the Patient Health Questionnaire-8 [23,24]. In order to assess baseline fitness and sports activities, a questionnaire was developed in cooperation with the Department of Sports Medicine at the University Tuebingen. The questionnaire contains questions concerning the individual fitness histories and baseline activity levels of individual patients in the form of patient-reported outcome measurement–related questions. The questionnaire includes 18 questions and is based on the previous work of the Department of Sports Medicine at the University Tuebingen and other groups [25,26].

### Translational Subproject

To further expand our knowledge on the possible pathomechanisms behind CRF and the potential effects that exercise has on the course and severity of CRF, blood samples will be taken from patients before radiotherapy, at the end of radiotherapy, and at 6 months after radiotherapy for the evaluation of inflammatory markers, such as neutrophil counts and interleukin-6 and C-reactive protein levels.

### Follow-up

Based on patients’ preferences, patients can keep the activity tracker after the completion of treatment. Independent of this decision, patients will be seen for follow-up at 3, 6, and 12 months after the completion of radiotherapy. Thereafter, follow-ups will take place yearly for up to 5 years.

### Sample Size and Statistical Considerations

Previous studies were able to demonstrate a medium to high effect size with regard to the influence that physical activity has on the fatigue and HRQoL of patients with cancer. Based on a medium effect size (Cohen *f*=0.25) with an α error of 5% and a power of 80%, a one-way independent sample analysis of variance was conducted. Per the results of this analysis, 201 patients (67 patients per arm) will be recruited for each trial [27]. A dropout rate of 20% was assumed for the calculation of the number of patients. Randomization will be carried out via block randomization with variable block lengths.

### Data Safety and Confidentiality

It will not be possible to record the position of patients in terms of their geographical location via the activity tracker. The clinical and personal data of patients will not be stored on the device. The assignment of the fitness trackers to the patients will be pseudonymized. The storage of fitness data and the linking of patient-related data will only be carried out within the hospital's information technology system. Therefore, these data will be subject to the hospital's data protection and data security regulations. For the purpose of publication, all data will be presented anonymously.

## Results

Both studies have been approved by the Institutional Review Board of University Clinic Tuebingen (OnkoFit I trial reference number: 201/2020BO2; OnkoFit II trial reference number: 202/2020B02) and are registered on ClinicalTrials.gov (OnkoFit I trial number: NCT04506476; OnkoFit II trial number: NCT04517019). The recruitment of patients started on August 1, 2020. The results will be published in a peer-reviewed journal upon the completion of the trial. As of June 2021, we have recruited 49 patients in the OnkoFit I trial and 12 patients in the OnkoFit II trial. Per our recruitment plan, 50 patients are expected to be recruited in both OnkoFit I and OnkoFit II trials per year. The reasons for the more rapid recruitment in the OnkoFit I trial than in the OnkoFit II trial are manifold. One reason is the prioritization of the OnkoFit I trial over the OnkoFit II trial during the COVID-19 pandemic. Another reason is the decline of the number of patients who are eligible for the OnkoFit II trial during the pandemic. However, we expect that the recruitment rate in the OnkoFit II will accelerate soon.

## Discussion

### Trial Implications

The benefit of physical activity both during and after various kinds of cancer treatments has been shown in several randomized trials. Physical activity not only improves HRQoL but also has the capability to reduce the severity of negative treatment effects. With regard to patients undergoing radiotherapy, the feasibility and positive impact of combining radiotherapy with physical activity has been observed, and further clinical studies are currently being conducted (Exercise Therapy in Radiation Therapy [EXERT] trial; NCT03905356) [28]. However, measured variables as well as definitions of end points greatly vary between different studies on using activity trackers in cancer care, and there is a great need for randomized trials that clarify optimal time points as well as strategies [24]. The rationale behind the OnkoFit trials is to test a physical activity program that requires only few resources and is easy to implement even in the workflow of departments that treat a very large numbers of patients (≥1000 patients per year). Even though there is no doubt that a one-to-one supervised physical activity program that is conducted over several weeks would be desirable, this is often not feasible due to costs, limitations in geographical reach, and a lack of qualified personal [29]. Moreover, in a qualitative interview study conducted by Hardcastle et al [30], survivors of breast cancer expressed that they would favor home-based programs for exercise. The high acceptance of activity trackers among patients with cancer has been reported previously [31-33]. In our pilot study, which investigated the feasibility of continuous activity monitoring, 19 of 23 patients regularly used a commercially available activity tracker during radiotherapy. In the same study, we observed very plausible results regarding changes in physical activity during the course of treatment [33]. These results are also supported by a study conducted by Ohri et al [34], who showed a correlation between decreasing step counts and a forthcoming need for the inpatient treatment of patients with lung cancer during radiotherapy.

We see several advantages in an activity tracker–based training program. First, most activity tracker devices are easy to use and can be intuitively used by most older patients without any previous experience with such devices. Second, these devices provide real-time feedback to patients, which results in self-awareness and motivation. Third, the quantitative measures of physical activity levels can be remotely shared with caregivers, thereby providing an objective view on a patient’s constitution and whether activity goals have been met [17]. The OnkoFit I and II trials have been designed as 3-arm trials. By introducing an arm in which patients receive an activity tracker but no predefined goals, we hope to study whether patients in the interventional arm, which provides weekly targets, actually have a higher level of physical activity and whether any potential effects that are observed at the end of the study are associated with the intervention.

Since acute and long-term side effects vary widely between patients with breast cancer undergoing radiotherapy and patients receiving preoperative or definitive radiotherapy, different end points were defined for the OnkoFit I and OnkoFit II trials. Additionally, the time points for assessment vary. In the OnkoFit II trial, the end point will be assessed 6 months after the end of radiotherapy and therefore later than in the OnkoFit I trial (the assessment will be conducted at 3 months), since patients receiving preoperative radiotherapy may still be in reconvalescence from surgery 3 months after the end of radiotherapy.

Patients undergoing radiochemotherapy in a neoadjuvant setting are at risk of increased side effects during and after concomitant surgery if they experience (severe) toxicity after the completion of radiation treatment. The concept of prehabilitation, which is defined as a rehabilitation program that is initiated before treatment, has gained more and more recognition in the field of oncology in recent years [35]. In two studies on colorectal patients, a prehabilitation program markedly improved functional recovery after resection, thereby underscoring the program’s potentially large benefit for patients [36,37]. In the context of neoadjuvant treatment, a study is currently underway for evaluating the effect that a concomitant prehabilitation program (conducted during chemotherapy) has on treatment outcomes and morbidity in patients with ovary cancer [38]. Similarly, another group has evaluated the effect that a prehabilitation program has on functional outcomes, particularly swallowing and the quality of life among patients undergoing radiochemotherapy for head and neck cancer [39]. Available data have been recently summarized by Squires and colleagues [40], with an emphasis on cardiovascular health after cancer therapy. In the context of the OnkoFit II trial, we aim to investigate whether the proposed fitness tracker–based exercise program can function as a prehabilitation program for patients undergoing neoadjuvant radiotherapy and thus decrease the number of postoperative complications.

One of the limitations of the proposed study is the lack of a supervised preintervention and postintervention fitness test (eg, ergometry), which would provide a more objective perspective on the fitness levels of patients than the fitness questionnaire—the one that will be used in our trials—alone. Furthermore, the trials were designed as monocenter trials that will be conducted at a single institution, which might limit the generalizability of the data.

### Conclusion

The randomized OnkoFit I and II trials will investigate a unique approach to conducting a patient-executed fitness program during and after radiation therapy. In these trials, activity trackers will be used to improve HRQoL; reduce the severity of treatment side effects, including CRF; and improve the preoperative health status of patients. The findings of these trials will help to further our knowledge on combining exercise therapy with radiation treatment that focuses on the patient point of view.

## Abbreviations

CRF: cancer-related fatigue
EXERT: Exercise Therapy in Radiation Therapy
FACIT-F: Functional Assessment of Chronic Illness Therapy-Fatigue
HRQoL: health-related quality of life
PRO-CTCAE: Patient-Reported Outcomes Common Terminology Criteria for Adverse Events
